# Evaluation and diagnosis of obstacles to land-based ecological security in resource-based cities: A case study of Xingtai city

**DOI:** 10.1371/journal.pone.0241618

**Published:** 2020-11-06

**Authors:** Chen Zhu, Yuping Li, Luxuan Zhang, Yanchao Wang

**Affiliations:** 1 College of Resources and Environment, Xingtai University, Xingtai, China; 2 University Malaysia Sabah, Kota Kinabalu, Malaysia; University of Defence, SERBIA

## Abstract

To provide a theoretical basis for sustainable land resource utilization and a reference for areas with similar natural conditions, an evaluation index for land-based ecological security was constructed based on the Driving force-Pressure-State-Impact-Response (DPSIR) model and the improved analytic hierarchy process (IAHP) and entropy methods, and the land-based ecological security status of Xingtai city from 2006 to 2017 was evaluated. Then, the obstacles to land-based ecological security were diagnosed. The results show that the values of the comprehensive evaluation index of land-based ecological security were 0.28–0.66 in the period from 2006 to 2017. The value of the index of land-based ecological security was low in the first seven years and gradually improved in the last five years of the study period. However, the overall situation was grave, and the ecological security conditions were poor. The main obstacles to land-based ecological security were the usage of pesticides, investment in environmental pollution treatments, the degree of machine cultivation, the rate of cultivation and the usage of fertilizer in Xingtai city. Based on the results of the land-based ecological security evaluation and the main obstacles identified in Xingtai city, this paper proposes management strategies and suggestions for improving land-based ecological security in Xingtai city. The specific proposals are as follows: vigorously develop green agriculture, increase investment in environmental pollution control, increase input in science and technology, and strengthen supervision and management of land use.

## 1 Introduction

The concept of land-based ecological security originated in research into ecological security. Ecological security is the cornerstone of the sustainable development of human society and an important condition for sustainable development [1]. In the early 1980s, Lester R. Brown, an American environmental scientist, first proposed the concept of ecological security and used it to describe the regional environment [2]. In the late 1980s, some scholars and institutions in the United States proposed that ecological security is a state in which the living environment of human beings is healthy and comfortable and in which social and economic development is stable and is not threatened by nature, society or other factors [3]. In the 1990s, the conflicts between humans and nature increased dramatically with rapid socioeconomic development. Research on ecological security has continued in many countries and has focused on the deterioration of the environment and the impacts of human productivity and livelihoods on the environment [4, 5]. With the development of this research, the concept of ecological security has gradually expanded to include the topic of land. The evaluation of land-based ecological security is the core of this concept, and it is an important basis for identifying early warnings as well as constructing and optimizing land-based ecological security patterns. The research on these evaluations mainly focuses on the evaluation index and the evaluation method. In indexes of evaluation, the main focus is on ecosystem health assessment, land quality assessment and land sustainability evaluation. In terms of the method of evaluation, the system decomposition method has been widely used in the field of land-based ecological security evaluation and research in recent years. The main idea of this method is that the land ecosystem, as a complete composite system, can be decomposed into several subsystems. The Pressure-State-Response (PSR) framework model is the most representative system decomposition scheme and is jointly proposed by the World Bank, the United Nations Development Agency (UNDP), the United Nations Food and Agriculture Organization (FAO), and the United Nations Environment Programme (UNEP). The main purpose of the model is to link land quality indicators and related policies and management decisions by using the impacts of land quality and the response of society to these pressures In addition, on the basis of the PSR model, the Driving force-State-Response (DSR) and the DPSIR framework systems have been proposed and revised by the United Nations Commission on Sustainable Development and the European Environment Agency [6, 7]. These framework systems mainly assess the relationship between human society and the ecosystem from the perspective of systems theory and have become a general framework in the field of land ecological evaluation. At the end of the 20th century, with the rapid development of urbanization and industrialization in China, the contradictory demands of humans and land had become increasingly prominent. The problem of land-based ecological security has been widely studied by academics and the public. The evaluation of land-based ecological security has become a key subject for experts and scholars. Some scholars have constructed an evaluation index system of land-based ecological security using different indicators from nature, economy and society and have carried out empirical analyses of land-based ecological security in different regions in China [13, 14] and obtained good research results. The PSR model is a commonly used land-based ecological security evaluation index system [8, 9] because the PSR framework can reflect the impact of human beings on the ecological environment, but its system structure is not ideal in terms of its indicators. Given the characteristics of land resources in China, the DPSIR has been widely recognized as a useful system for constructing a land-based ecological security evaluation index [10].

In recent years, the methods to determine index weight of each element in the evaluation index system involved the traditional methods (such as analytic hierarchy process (AHP) [11, 12] and entropy method) and new methods (such as best-worst method (BWM) [13] and full consistency method [14]). All of these methods can reflect the importance of the evaluation index to some extent but all are of some limitations. To better reflect the rationality of the evaluation index weight, the combed of the AHP and entropy methods were widely accepted by scholars due to the integrated characteristics of the subjective and objective evaluation method. The first reason is that the combed method can avoid some problems, such as more iterative times, a mass of calculations, and less or no consistency consistent of the judgment matrix in the AHP method. On the other hand, it prevents data inconsistencies between the actual and estimate value from the AHP and entropy methods.

Due to the differences in natural endowment and economic status among different regions, the core influencing factors of land-based ecological security are different in different region. Therefore, a more effective method can be selected to carry out environmental protection and governance for land, and only the main factors influencing land-based ecological security in a region need to be accurately understood. Some studies on the driving factors of land-based ecological security are mainly based on the impacts on ecological security from the perspective land-use change. Some scholars have performed many studies on domestic land-based ecological security [10, 15, 16]. However, the research on the evaluation and diagnosis of obstacles to land-based ecological security in Xingtai city have not been reported. During the rapid development of resource-based cities, serious environmental problems (such as serious pollution from solid, liquid, and gaseous wastes; large amounts of land occupation and destruction; low reclamation rates and so on) have developed in Xingtai. These problems have become a major obstacle to creating an ecological city and seriously affect the sustainable development of Xingtai city. It is very important to have a sensible view on land-based ecological security and assess land-based ecological security and diagnose its obstacles. Therefore, based on the DPSIR model, the IAHP, the entropy method, and the obstacle factor diagnosis method, this paper intends to construct an evaluation index system, evaluate the land-based ecological security situation, and distinguish the influencing factors for the land-based ecological security situation in Xingtai city. Furthermore, corresponding countermeasures and suggestions are put forward for the ecological security of land in Xingtai city, which provides a theoretical basis for the sustainable use of land and a reference for areas with similar natural conditions to those of Xingtai city.

## 2 Materials and methods

### 2.1 Overview of the study area

Xingtai city is located in southern Hebei Province (36°50′N-37°47′N, 113°52′-115°49′E), adjacent to Shandong Province in the east, Shanxi Province in the west, Handan city in the south, and Shijiazhuang city and Hengshui city in the north. The land area of this area is 12433 km^2^. It is an area with complex terrain, and the topography slopes from high to low from west to east. To the west is the Taihang Mountain area, the central area is hilly, and to the east is the North China Plain; the ratio of these areas is approximately 2:1:7. Xingtai city is not only an important coal base in China but it is also a new industrial base based on equipment manufacturing, the coal-salt chemical industry, energy generation, the automobile industry and the new building materials industry. It is a typical resource-based city and is also an important base for grain and cotton cultivation as well as livestock and poultry breeding. In 2017, the total population of the city was 7.899 million, and the cultivated land area was 691600 hm^2^. In recent years, due to the relatively large population and the rapid economic development, the relationship between people and land has become tense. The area of cultivated land has decreased sharply, the area of urban and rural residential land is too large, the productivity of the agricultural land is low, the proportion of forest (fruit orchard) land is relatively lower, the environment is fragile, and other land-based ecological security problems have become increasingly prominent; these problems seriously threaten the sustainable development of Xingtai city [17, 18].

### 2.2 Data sources

The data in this paper are from the Xingtai City Environmental State Bulletin [19], the Xingtai City Statistical Yearbook [18], the Hebei Economic Yearbook [20, 33] and the China Statistical Yearbook [21].

### 2.3 Research methods

In a comprehensive index evaluation system, the index weights are determined by subjective and objective weighting methods. The subjective weighting method determines the weight according to the subjective experience of the evaluator, such as in the scholar and expert scoring method. The weight based on the objective weighting method comes from objective reality and determines the index weights according to the inherent trends in the data for each index and the amount of information. The method selection is mainly determined by the availability of the data, the nature of the data and the size of the sample. If the data sample is small and the inherent trend in the data is not clear, it is appropriate to adopt the subjective weighting method; if the data sample is sufficient and has an obvious inherent trend, the objective weighting method is used. Based on the characteristics of the two methods and the actual local situation, this study combines the subjective and objective weighting methods to comprehensively determine the index weights [22].

#### 2.3.1 Improved analytic hierarchy process (IAHP)

The quantitative evaluation value of the judgment matrix in a traditional analytic hierarchy process (AHP) is evaluated on a 1 to 9 scale as proposed by T.L. Saaty [23]. When there are many indexes in the evaluation system, not only are the number of iterations and the computation requirements high but the consistency of the judgment matrix is also likely to be poor or even inconsistent, which leads to low reliability in the calculated weight. In this paper, the IAHP was employed at 3 scales (0, 1, 2). By pairwise comparison of the indexes, the comparison matrix was established, and then the judgment matrix was determined. The meaning of the 3 scale levels in the IAHP is as follows: 0 means that *X*_*i*_ is not as important as *X*_*j*_, 1 means that *X*_*i*_ and *X*_*j*_ are equally important, and 2 means that *X*_*i*_ is more important than *X*_*j*_. This method is self-regulating and does not require a consistency test. Compared with the traditional AHP, this method has a higher calibration value and better judgment transitivity. The decision maker improves the accuracy of the method in the process of comparative judgment [17, 24].

#### 2.3.2 Entropy method

Information entropy, which is based on the concept of thermodynamic entropy, is a measure of the disordered state of the system, i.e., the degree of uncertainty about the state of the system. It is generally believed that the greater the degree of variation in an index value is, the lower the information entropy, the more unbalanced the system structure, and the greater the amount of information provided by the index and the weight of the index, and vice versa. Therefore, the weight can be calculated according to the entropy value, that is, the variation degree of each index value [25–27]. The entropy method is an objective weighting method, which determines the index weight based on the information provided by the amount observed value.

## 3 Results and analysis

### 3.1 Construction of the evaluation index system and data standardization

Based on the concept of land-based ecological security, the actual situation in Xingtai city and the relevant references [11, 12, 28, 29], the evaluation system for land-based ecological security was divided into three layers: a target layer, a criterion layer, and an index layer. Based on the principles of accessibility, independence, pertinence and comparability of indicators, we selected 24 indexes that reflect driving forces, pressures, states, influences and responses to build the evaluation index system for land-based ecological security (Table 1).

**Table 1:** Land-based ecological security evaluation index system for in Xingtai city.

Target layer	Criterion layer	Indicator layer			
		Index layer meaning	code	Safety tendency	Index Description
Land ecological security (T)	driving (D)	Per capita GDP (yuan / person)	D_1_	+	GDP/ total population
		Population density (person / km^2^)	D_2_	-	Total population/ total land area
		Natural population growth rate (%)	D_3_	-	The data come from the statistical yearbook.
	pressure (P)	Economic density (yuan / km^2^)	P_1_	+	GDP/ Land area
		Proportion of primary industry (%)	P_2_	-	The data come from the statistical yearbook.
		Proportion of secondary industry (%)	P_3_	-	The data come from the statistical yearbook.
		Fertilizer usage (t/hm^2^)	P_4_	-	Total fertilizer application / cultivated land area
		Pesticide usage (kg/hm^2^)	P_5_	-	Total pesticide use / cultivated land area
		Urbanization level %	P_6_	-	The data come from the statistical yearbook.
		Per capita cultivated land (hm^2^/ person)	P_7_	+	Arable land area / total population
		Per capita water resources (m^3^/ person)	P_8_	+	Total water resources / total population
	status (S)	Employment rate (%)	S_1_	+	The data come from the statistical yearbook.
		Output value of GDP per unit cultivated land (yuan/hm^2^)	S_2_	+	The data come from the statistical yearbook.
		Proportion of grassland area (%)	S_3_	+	Grassland area / land area
		Proportion of forest land area (%)	S_4_	+	Forest land area / land area
		Forest coverage (%)	S_5_	+	The data come from the statistical yearbook.
		Land reclamation rate (%)	S_6_	-	The area of cultivated land / land area
		Grain output per unit cultivated land (kg/hm^2^)	S_7_	+	The data come from the statistical yearbook.
	influence (I)	Proportion of tertiary industry (%)	I_1_	+	The data come from the statistical yearbook.
		The degree of machine cultivation (%)	I_2_	+	Machine tillage area / cultivated land area
		Investments of environmental pollution treatment (%)	I_3_	+	Investment in Environmental pollution Control / GDP
	respond (R)	Soil and water coordination degree (%)	R_1_	+	Effective irrigation area / cultivated land area
		Level of farming mechanization (kw/hm^2^)	R_2_	+	Total power of agricultural machinery / cultivated land area
		Agricultural power intensity (kw·h/hm^2^)	R_3_	+	Rural electricity consumption / cultivated land area

To avoid the unpredictable influence of dimensional differences and make the data comparable, the data were converted into appropriate dimensionless indexes by range transformation, and the values of each index were converted into 0 to 1.

$$\text{Positive}\;\text{indicators}:Y_{ij}=\frac{X_{ij}-min(X_{ij})}{\text{max}(X_{ij})-\text{min}(X_{ij})}\;(i=1,2\ldots \text{n};j=1,2\ldots \text{m})$$ (1)

$$\text{Negative}\;\text{indicators}:Y_{ij}=\frac{\text{max}(X_{ij})-X_{ij}}{\text{max}(X_{ij})-\text{min}(X_{ij})}\;\;\;\;(i=1,2\ldots \text{n};j=1,2\ldots \text{m})$$ (2)

where *Y*_*ij*_ is the data matrix of the dimensionless indexes, *X*_*ij*_ is the original data matrix of the 24 evaluation indexes for Xingtai city from 2006 to 2017, *i* is the indicator (*i* = 1, 2… *n*), and *j* is the year (*j* = 1, 2… *m*). For positive security trend indexes, the larger the value, the more secure the land ecological state is, and Formula (1) was adopted for the standardization treatment. For negative security trend indexes, the larger the value, the less secure the land ecological state is, and Formula (2) was adopted for standardization.

### 3.2 Calculation of the land-based ecological security value

#### 3.2.1 Improved hierarchical weight

Following the weighting steps of IAHP, we invited some experienced experts and scholars to judge the relative importance of the evaluation indexes to the established land-based ecological security evaluation system. Based on the judgment results from the experts and scholars, a comparison matrix for the land-based ecological security in Xingtai city was obtained by calculation and collation (Tables 2–7). The individual and total hierarchical rankings for each land-based ecological security index are shown in Table 8, and the weight of each index is *M*_*i*_. The calculation steps are as follows:

①A relative importance judgment matrix G was determined at 3 scales (0, 1, 2) based on the judgment results from the experts and scholars
  $$G={\left(G_{ij}\right)}_{n\times n}$$ (3)
②The optimal transfer matrix B for target layer to criterion layer can be presented as follows
  $$B_{ij}=\frac{1}{n}\sum_{k=1}^{n}\left(T_{ik}+T_{kj}\right)$$ (4)
③The judgment matrix Q is expressed as:
  $$Q_{ij}=\text{exp}(B_{ij})$$ (5)
④The priority weights of these factors were calculated according to the judgment matrix Q. The eigenvector, corresponding to the maximum eigenvalue (λ_max_) of the judgment matrix Q, can be as relative weight value for each element in the layer and can be calculated with Formula (6).
  $$QC=\lambda_{max}C$$ (6)
  where C = [C_1_, C_2_, ⋯ C_n_]^T^ is the eigenvector matrix. The product square method was used to calculate the approximations of the eigenvectors.
  $$\{\begin{array}{c} C={\left[C_{1},C_{2}\ldots C_{n}\right]}^{T}\\ C_{i}={\left(\prod_{k=1}^{n}Q_{ij}\right)}^{\frac{1}{n}}/{\sum_{k=1}^{n}\left(\prod_{k=1}^{n}Q_{ij}\right)}^{\frac{1}{n}} \end{array}$$ (7)
⑤From the above results at the same level, the weights of all elements at this layer corresponds to the preceding layer can be calculated.
  $${\text{M}}_{\text{i}}=BL_{i}\times BP_{ij}$$
  where BL_i_ and BP_ij_ represents the weight value of the criterion layer and indicator layer, respectively.

**Table 2:** Comparison matrix of land-based ecological security indicators.

T-DPSIR	D	P	S	I	R
D	1	0	0	0	0
P	2	1	2	2	2
S	2	0	1	0	1
I	2	0	2	1	2
R	2	0	1	0	1

**Table 3:** Comparison matrix of driving forces.

D-D_1_D_2_D_3_	D_1_	D_2_	D_3_
D_1_	1	1	2
D_2_	1	1	1
D_3_	0	1	1

**Table 4:** Pressure comparison matrix.

P-P_1_P_2_P_3_P_4_P_5_P_6_P_7_P_8_	P_1_	P_2_	P_3_	P_4_	P_5_	P_6_	P_7_	P_8_
P_1_	1	0	0	0	0	1	0	0
P_2_	2	1	2	0	0	0	1	2
P_3_	2	0	1	0	0	1	0	0
P_4_	2	2	2	1	1	2	2	2
P_5_	2	2	2	1	1	2	2	2
P_6_	1	2	1	0	0	1	2	1
P_7_	2	1	2	0	0	0	1	1
P_8_	2	0	2	0	0	1	1	1

**Table 5:** State comparison matrix.

S-S_1_S_2_S_3_S_4_S_5_S_6_S_7_	S_1_	S_2_	S_3_	S_4_	S_5_	S_6_	S_7_
S_1_	1	0	0	0	0	0	0
S_2_	2	1	2	0	0	1	1
S_3_	2	0	1	1	1	0	0
S_4_	2	2	1	1	1	2	2
S_5_	2	2	1	1	1	1	2
S_6_	2	1	2	0	1	1	1
S_7_	2	1	2	0	0	1	1

**Table 6:** Impact comparison matrix.

I-I_1_I_2_I_3_	I_1_	I_2_	I_3_
I_1_	1	0	0
I_2_	2	1	0
I_3_	2	2	1

**Table 7:** Response comparison matrix.

R-R_1_R_2_R_3_	R_1_	R_2_	R_3_
R_1_	1	0	0
R_2_	2	1	1
R_3_	2	1	1

**Table 8:** Individual and total rankings of land-based ecological security index levels in Xingtai.

administrative levels	D	P	S	I	R	Weight (M_i_)
	0.0774	0.3834	0.1411	0.2570	0.1411	
D_1_	0.4484					0.0347
D_2_	0.3213					0.0249
D_3_	0.2302					0.0178
P_1_		0.0520				0.0199
P_2_		0.1102				0.0423
P_3_		0.0668				0.0256
P_4_		0.2332				0.0894
P_5_		0.2332				0.0894
P_6_		0.1102				0.0423
P_7_		0.0972				0.0373
P_8_		0.0972				0.0373
S_1_			0.0556			0.0078
S_2_			0.1310			0.0185
S_3_			0.0984			0.0139
S_4_			0.2319			0.0327
S_5_			0.2010			0.0284
S_6_			0.1511			0.0213
S_7_			0.1310			0.0185
I_1_				0.1483		0.0381
I_2_				0.2889		0.0742
I_3_				0.5627		0.1446
R_1_					0.1554	0.0219
R_2_					0.4223	0.0596
R_3_					0.4223	0.0596

#### 3.2.2 Entropy weight method

The calculation steps for the entropy weights [30] are as follows:

① The proportion of each index to the total of the indicators in each year was calculated as follows:
  $$P_{ij}=\frac{Y_{ij}}{\sum_{i=1}^{n}Y_{ij}}$$ (8)
② The entropy of each index was calculated as follows:
  $$E_{i}=-K\sum_{j=1}^{m}\left(P_{ij}\times lnP_{ij}\right)$$ (9)
  $$K=\frac{1}{lnm}$$
  When *P*_*ij*_ = 0, *P*_*ij*_ × *lnP*_*ij*_ = 0..
③ The weight of each index was calculated as follows:
  $$W_{i}=\frac{1-E_{i}}{n-\sum_{i=1}^{n}E_{i}}$$ (10)
  where *i* is the index (*i* = 1,2 … n), *j* is the year (*j* = 1,2 … m), *Y*_*ij*_ is the normalized value, *P*_ij_ is the proportion of each index to the sum of the indexes per year, *E*_*i*_ is the entropy value of the index, and *W*_*i*_ is the weight of the index. The results are shown in Table 9.

**Table 9:** Entropy weight of each index.

Index code	Entropy weight (*W*_*i*_)	Index code	Entropy weight (*W*_*i*_)	Index code	Entropy weight (*W*_*i*_)	Index code	Entropy weight (*W*_*i*_)
D_1_	0.0436	P_4_	0.0371	S_2_	0.0361	I_1_	0.0483
D_2_	0.0388	P_5_	0.0659	S_3_	0.0415	I_2_	0.0551
D_3_	0.0236	P_6_	0.0246	S_4_	0.0516	I_3_	0.0545
P_1_	0.0351	P_7_	0.0337	S_5_	0.0505	R_1_	0.0273
P_2_	0.0332	P_8_	0.0171	S_6_	0.0623	R_2_	0.0361
P_3_	0.0503	S_1_	0.0591	S_7_	0.0365	R_3_	0.0381

#### 3.2.3 Comprehensive weight

The weights from the IAHP and entropy weight method could both reflect the importance of each evaluation index to a certain extent, but they have some limitations. To better reflect the meaning of the weight of an evaluation index, we combined the characteristics of the two evaluation methods, and the values of the comprehensive evaluation weights were obtained by the arithmetic average (Eq 11). This method not only avoids the subjectivity of IAHP but also eliminates the inconsistency between the weight from the entropy method and the actual index. Finally, the comprehensive index weights *A*_*i*_ of the land-based ecological security rating system indexes are shown in Table 10.

$$A_{i}=\frac{W_{i}+M_{i}}{2}$$ (11)

**Table 10:** Comprehensive weight of the indexes.

Index code	Comprehensive weight (*A*_*i*_)	Index code	Comprehensive weight (*A*_*i*_)	Index code	Comprehensive weight (*A*_*i*_)	Index code	Comprehensive weight (*A*_*i*_)
D_1_	0.0392	P_4_	0.0633	S_2_	0.0273	I_1_	0.0432
D_2_	0.0318	P_5_	0.0777	S_3_	0.0277	I_2_	0.0647
D_3_	0.0207	P_6_	0.0334	S_4_	0.0422	I_3_	0.0996
P_1_	0.0275	P_7_	0.0355	S_5_	0.0394	R_1_	0.0246
P_2_	0.0377	P_8_	0.0272	S_6_	0.0418	R_2_	0.0479
P_3_	0.0379	S_1_	0.0335	S_7_	0.0275	R_3_	0.0488

#### 3.2.4 Land-based ecological security value

The model for the security value of a single land ecology index is as follows:

$$H_{i}=Y_{ij}\times A_{i}$$ (12)

In Eq (12), *H*_*i*_ is the value of the land-based ecological security, *Y*_*ij*_ is the standardized value of index *i* in year *j*, and *A*_*i*_ is the weight of index *i*. The security value of a single index can reflect the land-based ecological security situation in Xingtai city from different perspectives. However, to reflect the present land-based ecological security situation in this area well, it is also necessary to carry out a comprehensive calculation with the index. The mathematical model for the comprehensive value of land-based ecological security is as follows:

$$T_{j}=\sum_{i=1}^{n}\left(Y_{ij}\times A_{i}\right)$$ (13)

where *T*_*j*_ is the comprehensive value of land-based ecological security in year *j*, *i* is the index, *j* is the year, and *n* is the number of indexes. The calculated results are shown in Table 11.

**Table 11:** Land-based ecological security evaluation index values in Xingtai city from 2006 to 2017.

Year	Comprehensive land security evaluation value (*T*_*j*_)	Driving force evaluation value	Pressure evaluation value	State evaluation value	Influence evaluation value	Response evaluation values
2006	0.28	0.05	0.09	0.06	0.04	0.03
2007	0.37	0.05	0.10	0.10	0.04	0.08
2008	0.36	0.04	0.08	0.10	0.09	0.05
2009	0.35	0.04	0.18	0.06	0.05	0.02
2010	0.35	0.03	0.16	0.06	0.06	0.04
2011	0.37	0.04	0.16	0.08	0.04	0.05
2012	0.34	0.04	0.15	0.07	0.01	0.07
2013	0.50	0.04	0.22	0.12	0.04	0.08
2014	0.52	0.04	0.15	0.08	0.15	0.09
2015	0.48	0.05	0.16	0.10	0.08	0.10
2016	0.45	0.05	0.17	0.10	0.07	0.06
2017	0.66	0.06	0.22	0.15	0.15	0.07

#### 3.2.5 Evaluation criteria and grades of land-based ecological security

According to the comprehensive safety values, the research achievements and standards of China, the industry and the local government [22, 31, 32], and the background value of each index in the study area, we defined the comprehensive evaluation criteria for land-based ecological security in Xingtai city with expert consultation, public participation and other methods. The range of security synthesis values (0 to 1) is divided into five grades, and the system characteristics of the five levels are described in Table 12. The greater the comprehensive security value, the better the ecological security, and vice versa.

**Table 12:** Description of the land-based ecological security index value ranges and system characteristics.

safety index value	grade	characteristics
*T*_*j*_<0.85	I	The service function of the land ecosystem is completely functional; the system structure is intact; the environment is basically undisturbed; the regeneration capacity of the ecosystem is strong; the agricultural pollution, desertification, and alkalinization are low; the vegetation cover rate is high; ecological disasters are very rare; and the ecological problems are not obvious.
0.65<*T*_*j*_≤0.85	II	The service function of the land ecosystem is nearly completely functional; the system structure is relatively intact; the ecological environment is less damaged, and after a disturbance, it can generally be restored; the ecological problems are not significant; and ecological disasters are not significant.
0.55<*T*_*j*_≤0.65	III	The service function of land ecosystem has been degraded; the system structure has deteriorated; the ecological environment has been destroyed to some extent, but it can still maintain its basic functions, and it deteriorates readily after being disturbed; the ecological problems are obvious, and ecological disasters often occur.
0.35<*T*_*j*_≤0.55	IV	The service function of the land ecosystem is seriously degraded; the system structure is mostly destroyed; the environment has been damaged to a large extent, and the system has difficulty recovering after being disturbed by external factors; the ecological problems are significant; and ecological disasters occur more frequently.
*T*_*j*_≤0.35	V	The service function of land ecosystem has almost collapsed; the structure of the system is incomplete; the environment is seriously damaged; it is difficult to restore and reconstruct the system after external interference; and ecological disasters occur frequently.

### 3.3 Analysis of the results of the land-based ecological security assessment

The comprehensive land-based ecological security evaluation index from 2006 to 2017 in Xingtai city is shown in Table 11. The range of the comprehensive evaluation index values are between 0.28 and 0.66. The status of land-based ecological security in the first seven years is low, and there is a trend of gradual improvement in the next five years, but the overall situation is serious, and the security situation does not look promising. The minimum value of the index was calculated for 2006 and the maximum was calculated for 2017, at values of 0.28 and 0.66, respectively. According to the evaluation criteria and the grades of land-based ecological security (Table 12), *T*_*j*_≤0.35 in 2006, 2009, 2010, and 2012, and the land-based ecological security grade was V. The results showed that in these years, the service function of the land ecosystem almost collapsed, the structure of the system was incomplete, and the ecological environment was seriously damaged, which indicates that the restoration and reconstruction of the system after external interference was very difficult and that ecological disasters occurred frequently. In 2007, 2008, 2011, and 2013–2016, the land-based ecological security evaluation index value was between 0.36 and 0.52, in the range of 0.35<*T*_*j*_≤0.55. This land-based ecological security grade was IV. The results showed that in these years, the service function of the land ecosystem was seriously degraded and the system structure was destroyed. The ecological environment had been damaged to a great extent, and the system had difficulty recovering after an external disturbance. Moreover, the ecological problems were large, and the ecological disasters were more common. In 2017, the land-based ecological security evaluation index value was 0.66, which was the maximum during the evaluation period. In the range of 0.65<*T*_*j*_≤0.85, the land-based ecological security grade was II. The service function of the land ecosystem was relatively functional, and the system structure was relatively intact. The environment was less damaged, and there were fewer significant ecological and disasters.

### 3.4 Diagnosis of obstacles

After a comprehensive evaluation of land-based ecological security, it is also necessary to analyze and diagnose the obstacles that affect the level of land-based ecological security to land-based ecological security problems [33–35]. Three variables, the factor contribution, index deviation and obstacle degree, were introduced to diagnose obstacles. The factor contribution degree represents the influence of a single index on the total goal, which is generally expressed by the weight *A*_*i*_ of the single index. The index deviation *N*_*ij*_ represents the difference between the value of the single index and the ideal target value and is expressed by the difference between the standardized value of the single index *Y*_*ij*_ and 100%. The obstacle degree *F*_*ij*_ indicates the degree to which each individual index represents an obstacle to land-based ecological security. The formula for the obstacle degree is shown below as Eqs (14) and (15), and the results are shown in Table 13.

$$N_{ij}=1-Y_{ij}$$ (14)

$$F_{ij}=\frac{N_{ij}*A_{i}}{\sum_{i=1}^{n}N_{ij}*A_{i}}$$ (15)

With Eqs (14) and (15), the obstacle degree land-based ecological security evaluation index was calculated for Xingtai city from 2006 to 2017. The results showed that the obstacles for land-based ecological security were dynamic rather than static in Xingtai city. The obstacles change in each year in terms of the categories of obstacles, the size of the obstacle degree and the ranking of obstacles. Because many indexes were selected for the evaluation system, this paper only lists the top five indexes of the obstacle degree in each year (Table 13) and provides the index value and rate of frequency (Table 14). Finally, the five most frequently occurring factors are the main obstacles that affect land-based ecological security (Table 14). During 2006–2017, the obstacles with the highest frequency of occurrence were pesticide usage, investments in environmental pollution treatment (measured as the environmental investment in proportion to the GDP), the degree of machine cultivation, cultivation rates and fertilizer usage. In the 12 years, the frequency of pesticide usage was 11, and the obstacle value was 9.62%-12.81%. The frequency of investment in environmental pollution treatment was 11, and the obstacle value was 6.14%-18.12%. The frequency of the degree of machine cultivation was 10, and the obstacle degree was 6.04%-10.98%. The frequency number of the rate of cultivation was 9, and the obstacle degree was 6.22%-10.58%. The frequency of fertilizer use was 6, and the obstacle degree was 5.56%-9.91%. According to the distribution structure of the obstacles in the criterion layer, among the top five obstacles, the usage of pesticide and fertilizer falls into the pressure layer, the investment in environmental pollution treatment and the degree of machine cultivation are part of the influence layer, and the rate of cultivation is part of the state layer. Based on the obstacle degree rankings, the investment in environmental pollution treatment ranked first in seven years, including 2006, 2010–2013, 2016 and 2017, and the usage of pesticide ranked first in the rest of the years, which were from 2007 to 2009, 2014 and 2015. The results showed that investments in environmental pollution treatment are the most important obstacle affecting land-based ecological security. How to coordinate development between economic growth and ecological construction is an urgent problem that needs to be studied and solved. To improve the level of land-based ecological security in Xingtai city, the growth rate of the investment in environmental pollution treatment should remain consistent with the growth rate in the GDP. Meanwhile, it is necessary to increase the investment in environmental pollution treatment, strengthen environmental supervision, and increase financial support for environmental protection. The application of pesticides and fertilizers can increase grain production in the short term, but it generates a large land pollution load for the ecosystem and poses a threat to the security of land ecosystems in the long term. Because of the large agricultural population, the proportion of cultivated land area and the relatively underdeveloped farming methods, the land reclamation rate and the degree of machine cultivation have a great impact on regional land-based ecological security. To improve the level of land-based ecological security, agricultural practices will require significant attention. Based on the criterion layers, the obstacles to land-based ecological security in Xingtai city mainly come from the pressure layer and the influence layer.

**Table 13:** Main obstacles to land-based ecological security in Xingtai city.

year	Order of obstacle degree					
		1	2	3	4	5
2006	Obstacle facto	I_3_	P_5_	P_4_	R_3_	I_2_
	Impediments	10.38	9.62	7.12	6.76	6.38
2007	Obstacle factor	P_5_	I_3_	I_2_	P_4_	I_1_
	Impediments	11.27	11.02	9.80	7.49	6.09
2008	Obstacle factor	P_5_	P_4_	I_2_	R_3_	I_3_
	Impediments	12.16	9.91	7.02	6.96	6.14
2009	Obstacle factor	P_5_	I_3_	I_2_	R_3_	S_6_
	Impediments	9.82	9.70	9.19	6.88	6.32
2010	Obstacle factor	I_3_	P_5_	I_2_	S_6_	R_3_
	Impediments	11.48	10.61	6.34	6.32	5.73
2011	Obstacle factor	I_3_	P_5_	S_6_	I_2_	P_3_
	Impediments	15.41	11.31	6.62	6.04	5.26
2012	Obstacle factor	I_3_	P_5_	I_2_	S_6_	I_1_
	Impediments	14.89	11.36	9.86	6.22	4.67
2013	Obstacle factor	I_3_	I_2_	S_6_	P_4_	I_1_
	Impediments	18.12	10.59	7.93	5.56	5.39
2014	Obstacle factor	P_5_	P_4_	S_6_	I_2_	S_3_
	Impediments	12.81	9.01	7.87	7.65	5.73
2015	Obstacle factor	P_5_	I_3_	I_2_	P_4_	S_6_
	Impediments	11.56	11.36	10.98	8.43	7.37
2016	Obstacle factor	I_3_	P_5_	R_2_	S_6_	P_7_
	Impediments	18.04	10.73	8.68	6.90	6.05
2017	Obstacle factor	I_3_	R_2_	P_5_	S_6_	P_7_
	Impediments	15.57	12.77	11.59	10.58	10.5

**Table 14:** Frequency of main obstacles to land-based ecological security in Xingtai city.

Obstruction factor	P_3_	P_4_	P_5_	P_7_	S_3_	S_6_	I_1_	I_2_	I_3_	R_2_	R_3_
Frequency (times)	1	6	11	2	1	9	3	10	11	2	4
Frequency (%)	8.33	50.00	91.67	16.67	8.33	75.00	25.00	83.33	91.67	16.67	33.33

## 4 Conclusion and discussion

Based on the DPSIR model, the IAHP and entropy methods are used to evaluate the land-based ecological security in Xingtai city from 2006 to 2017, and the impact factors for land-based ecological security are diagnosed. The results show that the comprehensive evaluation index of land-based ecological security varied from 0.28 to 0.66 from 2006 to 2017 in Xingtai city, and the values were in the II, IV and V categories with 1, 7 and 4 years, respectively. The level of land-based ecological security was low in the first seven years and gradually improved in the last five years, but the overall situation was concerning, and the ecological security situation was not promising. According to the obstacles for the index layer, the main obstacles to land-based ecological security are the usage of pesticides, investments in environmental pollution treatment, the degree of machine cultivation, the rates of cultivation and the usage of fertilizer in Xingtai city. On the basis of the position of the obstacle degrees of the criterion layers, the obstacles to ecological security in Xingtai city mainly come from the pressure layer and the influence layer. In addition, it is worthwhile to note that the comprehensive evaluation index series in our study showed an similar trend to that in Hebei province [36], with a significantly and positively correlation coefficients 0.839 (n = 8, p = 0.009, 2010–2017). These results indicated that the land-based ecological security in Xingtai city is improving with the serious situation, and it needs to be further raised. With the enhancing awareness of environment protection and the increasing proportion of investment in environmental pollution control, the land ecological environment will gradually improve.

Based on the land-based ecological security evaluation results and the main obstacles identified in Xingtai city, this paper proposes the following countermeasures to guarantee land-based ecological security in Xingtai city. First, we should vigorously develop green agriculture, accelerate scientific and technological innovations in agriculture, rationally apply pesticides and fertilizers, reduce the pollution of cultivated land resources, and strengthen and improve the norms and regulatory systems surrounding the use of pesticides and fertilizers in order to decrease fertilizer and pesticide use [33–35]. Second, the investment in environmental pollution treatments should be increased, the growth rate of investments in environmental pollution treatment should keep pace with the GDP growth rate, the supervision of treatment implementation should be strengthened, and financial support for land ecological construction should be increased to ensure land-based ecological security in Xingtai city. Third, investments in science and technology should be increased, underdeveloped farming methods should be changed, and mechanization should be increased. Fourth, land use supervision and management should be strengthened, rural land restoration should be actively carried out, and the intensity of land use should be increased.

Many factors influence land-based ecological security. Due to restrictions on data availability, only 24 indexes were selected for the construction of the evaluation system, which brought subjectivity into the selection of the land-based ecological security index and the determination of evaluation criteria. Therefore, it is necessary to construct a scientific, comprehensive, objective and reasonable evaluation system for further research. The IAHP was employed to improve the scale of the evaluation method as it is more flexible, rapid and easy to use than other methods. In addition, we combined the IAHP with the entropy method to determine the weights in a more scientific and reasonable way. However, with the continuous progress in science and technology, research methods such as landscape ecology principles, neural networks, 3S technology and other methods have gradually improved, and new methods and new models are continuously being introduced. How to develop new research methods will also become one of the hotspots of land-based ecological security research in the future.

## Supporting information

S1 File (XLSX)
